# The effect of *Lactobacillus reuteri* supplementation in *Helicobacter pylori* infection: a placebo-controlled, single-blind study

**DOI:** 10.1186/s40795-018-0257-4

**Published:** 2018-12-07

**Authors:** Martin Buckley, Sean Lacey, Andrea Doolan, Emily Goodbody, Kelly Seamans

**Affiliations:** 1 Mercy University Hospital, Grenville Place, Centre, Cork, T12 WE28 Ireland; 2 Cork Institute of Technology, Rossa Avenue, Bishopstown, Cork, T12 P928 Ireland; 3 Atlantia Food Clinical Trials, Heron House Offices First Floor, Blackpool Retail Park, Cork, T23 R50R Ireland

## Abstract

**Background:**

*Helicobacter pylori* is the major cause of chronic gastritis, and considered as a risk factor for peptic ulcer and gastric cancer. The *H. pylori* standard antibiotic therapy fails in about 25–30% of cases, particularly because of the increasing occurrence of resistance to antibiotics. The aim of the current study was to investigate whether the strain *Lactobacillus reuteri* DSM17648 which has been previously shown to reduce *Helicobacter pylori* load additionally improves gastrointestinal symptoms in *H. pylori* positive subjects when used in a 28 days supplementation.

**Methods:**

In a single-blinded, placebo controlled study 24 *H. pylori*-positive adults (13 females, 11 males; median age: 43.5) with mild dyspepsia (mean GSRS score: 11.82) received placebo for 28 days followed by Pylopass™ containing the *L. reuteri* DSM 17648 (2 × 10^10^ cells per day) for the following 28 days. After 28 days of Pylopass*™* supplementation the change in *H. pylori* load was measured by ^13^C urea breath test (^13^C-UBT) and the change in symptoms were determined by the Gastrointestinal Symptom Rating Scale (GSRS). In addition, blood assessments were conducted to measure the physiological changes relevant in terms of safety.

**Results:**

After a 28-day supplementation phase with Pylopass*™* there was a trend for reduction of *H. pylori* load in 62.5% of the subjects and for the overall GSRS scores in 66.7% of subjects. The overall GSRS scores from baseline to day 56 following all 24 subjects undergoing the placebo phase followed by the Pylopass™ phase was significantly decreased (*p* = 0.005). The mean 13C-UBT δ value decreased by 22.5% during the Pylopass™ supplementation phase (− 3.14), while the mean 13C-UBT δ increased by 37.3% (+ 3.79) in the placebo phase. No side effects were reported in either study phase.

**Conclusion:**

The results demonstrated that *L. reuteri* DSM17648 has the potential to suppress *H. pylori* infection, and may lead to an improvement of *H. pylori*-associated gastro intestinal symptoms. Further studies with adequate power should be performed.

**Trial registration:**

Clinicaltrials.gov: NCT02051348 (January 30, 2014).

## Background

*Helicobacter pylori* (*H. pylori*) is a widespread human pathogen that infects at least 50% of the global population causing gastric symptoms and leading to further disease in 20% of those infected. The prevalence of *H. pylori* infection differs between regions of the developing world (e.g. Southeast Asia; the Indian subcontinent; Latin America) where prevalence rate in adults is up to 80%, and industrialized nations, where the incidence is significantly less (20 to 50%) [1, 2]*.*

The Maastricht/Florence Consensus report, which outlines the diagnostic guidelines and treatment strategies for those with *H. pylori* [3] advises individuals with certain risk factors to undergo eradication therapy. In particular, it is recommended that those with functional dyspepsia, undergo the “test and treat” strategy. However, there remains a lack of options for volunteers who are either asymptomatic or experience only mild gastrointestinal symptoms or for patients that have unsuccessfully undergone the standard treatment due to *H. pylori* antibiotic resistances or those showing low compliance due to massive side effects of antibiotic treatment. Alternative anti-*H. pylori* treatments are searched for. The use of probiotics as monotherapy or, synergistically (in combination with antibiotics) is researched as an alternative way of controlling *H. pylori* infection and reducing side effects of antibiotic treatment [4–8].

Mechanisms by which probiotics work in this application include suppressive effects against gastrointestinal inflammation and against *H. pylori* [9]. Probiotics might enhance the production of prostaglandin, mucins, growth factors and anti-inflammatory cytokines, and can stabilize or strengthen the gut mucosal barrier [10–12]. Other mechanisms include production of antimicrobial substances [13] or displacement of *H. pylori* through competitive binding to adhesion receptors of *H. pylori* [14].

However, successful in vivo studies demonstrating the effects of probiotics on *H. pylori* gastritis are limited. This could be due to the adverse physiological conditions of the stomach, such as an acidic environment, gastric enzymes, bile acids and mechanical stress that reduce the survival and metabolic activity of probiotics. A study in 2015 [15] identified a specific *Lactobacillus reuteri* strain (DSM17648) that exhibits a novel mechanism of action against *H. pylori* which is working under harsh stomach conditions. *L. reuteri* DSM1768 cells suspended in a matrix and spray dried (Pylopass™) remain active as non-viable cell preparation. The strain acts against *H. pylori* in the stomach by specifically binding and co-aggregating *H. pylori.* Binding to *L. reuteri* masks surface structures of *H. pylori* and severely impedes its motility. The aggregated *H. pylori* no longer adhere to the gastric mucosa and the *Lactobacillus* / *Helicobacter* complexes are flushed out the stomach.

Through in vitro and human studies, *L. reuteri* DSM17648 has been shown to exert a significant lowering effect on the *H. pylori* load. Two human pilot studies have demonstrated that oral administration of *L. reuteri* DSM17648 (Pylopass™) leads to a reduction in UBT values in volunteers with *H. pylori* [15, 16]. The aim of this study was to measure the effect of Pylopass*™* supplementation over a longer period (4 instead of 2 weeks) and to assess the effect of *L. reuteri* DSM17648 on mild gastric symptoms associated with *H. pylori* infection.

## Methods

### Study population

A placebo-controlled, single-blind study of Pylopass™ containing *L. reuteri* DSM17648 versus placebo in subjects who are *Helicobacter pylori* carriers and show mild indigestion was conducted at the Clinical Trial Unit of University College of Cork and the Mercy University Hospital, Cork, Ireland. 115 healthy volunteers were screened for *H. pylori* infection using a ^13^C UBT test. Among them, 24 (13 female, 11 male) had positive results for *H. pylori* and were enrolled into the study. The sample size was calculated using the mean urea breath test values from baseline and post-test conditions as the standard deviation. The type I error rate (α) was assumed to be 0.05, and statistical power β = 0.20, it was estimated that the study required 20 subjects to achieve 80% power. To account for dropouts, an additional 20% was added to the *n* so that the total recruitment included 24 randomized volunteers. The subjects were between 18 and 75 years of age, with good general health or mild digestive discomfort (such as indigestion), a positive UBT (Helicobacter test δ > 1.5%) and had documented informed consent. Exclusion criteria were pregnancy/lactation, hypersensitivity to any of the components of the test product, any active gastrointestinal disorder or previous gastrointestinal surgery, significant acute or chronic condition or consumption of any medications (e.g. immunosuppressive drugs) deemed by the principle investigator to have the potential to confound the study or pose a safety risk, entry to the study, active gastrointestinal disorder or previous gastrointestinal surgery, condition or intake of any medication interfering with the objectives of the study, posing a safety risk or confounding the interpretation of the study results, intake of PPIs or gastroprotective medicines, oral intake of antibiotics in the past 3 months, prior eradication therapy with antibiotics, any major dietary changes in the past 3 months, intake of non-steroidal anti-inflammatory drugs (NSAIDs) within 2 weeks of baseline visit or for the duration of the trial, alcohol or drug abuse, participation at other clinical trials at the same time or completed not less than 60 days prior to this study and any surgical procedures, malignant disease or concomitant end-stage organ disease.

### Study supplements

The active supplement consisted of 100 mg Pylopass™ (1 × 10^10^ spray-dried cells of *Lactobacillus reuteri* DSM17648) (LONZA Group Ltd., Switzerland), FOS, Sorbitol (E420i), Xylitol (E-967), Flavor, Silicium oxide (E551), Magnesium stearate (E470b) and Sucralose (E955), (Eladiet S.L., Spain) prepared as solid tablets for oral application. The daily dosage of 2 tablets corresponds to 2 × 10^10^ cells. The Pylopass™ and placebo tablets were identical in weight (600 mg), size color and flavor. The test products were produced in compliance with the requirements for Good Manufacturing Practices for nutrition ingredients (Food GMP) (Eladiet S.L., Spain).

### Study design

The study was conducted according to the Declaration of Helsinki. All patients gave written informed consent prior to their participation in the study. The study protocol was approved by the local ethics advisory committee (Clinical Research & Ethics Committee of the Cork Teaching Hospitals, Cork, Ireland).

**Fig. 1:**
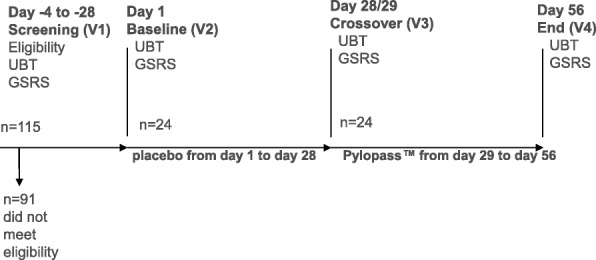
Study flow chart. Out of 115 subjects, 24 subjects met the inclusion criteria and were enrolled. All subjects completed the study. Subjects consumed placebo for 4 weeks (day 1 -day 28) and then changed to Pylopass™ containing *Lactobacillus reuteri* DSM17648 (2 × 10^10^ cells) from day 29 through day 56. They were blinded as to which product they were receiving

The study was a single-blind study. All participants began with placebo for the first 28 days, but were blinded to the product they received. During the weeks 5 through 8, all volunteers were provided with active supplement (Pylopass™), but were blinded to the product they received. An initial phone screen was performed, where subjects were asked questions regarding their age and general health. Eligible subjects were scheduled for a screening visit where demographic data, medical history and general health were recorded. Vitals, including weight, height, blood pressure and pulse were recorded, and the 13C-urea breath test (13C-UBT) was carried out. For women of childbearing age a pregnancy test was performed. After being included in the study subjects were instructed to take one tablet after breakfast and one tablet after their evening meal. The subjects completed the Gastrointestinal Symptom Rating Scale (GSRS) survey and a urease breath test was performed. A 16 ml fasting blood sample was collected to assess biochemical and haematological parameters, including lipid profile and blood glucose to test for physiological changes relevant in terms of safety. After the 28 days of supplementation with the placebo the second ^13^C-UBT was conducted, the GSRS was completed and the subjects were queried about any changes in their health status or medications and any adverse events were recorded. Then the subjects were provided with 28 days’ supply of Pylopass™ containing *Lactobacillus reuteri* DSM 17648. After 28 days’ supplementation, the subjects returned to the study site at day 56 for the last visit, where the GSRS was completed, the *H. pylori* load was reassessed by ^13^C-UBT, and the subjects were queried about any changes in their health status or medications and any adverse events were recorded (Fig. 1). Participants were instructed not to initiate any lifestyle or dietary changes throughout the duration of the study.

### Outcome measures

#### Helicobacter assessment 13C-urea breath test (UBT)

The detection of *H. pylori* infection in the screening phase for confirmation of eligibility and the quantification of colonization to measure the effects of Pylopass*™* supplementation was carried out by a breath test. The Urease breath test (Diabact UBT) is a rapid, non-invasive diagnostic procedure to assess the *H. pylori* infection status. The test is based upon the ability of *H. pylori* to convert urea to ammonia and carbon dioxide by urease activity. After an overnight fast, subjects swallowed urea labelled with non-radioactive carbon-13 (50 mg ^13^C-urea). Carbon dioxide resulting from the degradation of urea containing this isotope by *H. pylori* urease in the stomach is detectable by mass spectroscopy in the exhaled breath. As there is a small amount of naturally occurring ^13^C even in the absence of urease activity, breath samples are taken before and 10 min after the ingestion of ^13^C urea. The measurement considered *H. pylori* positive, if the difference (δ) in ^13^C/^12^C of 0-min-value and 10-min-value exceeds 1.5 ‰. If there is no difference, the test is negative, indicating no infection with *H. pylori*. All samples were analyzed in the Gastroenterology laboratory in the Mercy University Hospital which was accredited for the urease breath test. Three ^13^C urea breath tests were taken from the randomized subjects at each visit at the study site: baseline (day 1),end of placebo/start of Pylopass™ (day 28) and after completion of the Pylopass*™* supplementation (day 56). The breath test analyses were represented as change in ^13^C-UBT (ΔδUBT) calculated as absolute differences from baseline (day 1) to end of supplementation with placebo (day 28) and after application of Pylopass™ from day 29 to day 56 (V4). The Δplacebo and Δ*L. reuteri* values were calculated as means ± standard deviation (day 28 - day 1 and day 56 – day 28).

#### Symptom assessment (GSRS)

The changes in symptoms were recorded using the Gastrointestinal Symptom Rating Scale (GSRS) at baseline (day 1), at end of placebo phase (day 28) after application of placebo and after completion of Pylopass*™* supplementation at endpoint (day 56). 15 standardized questions were scored and summarized to 5 major categories: abdominal pain (Q1, 7 & 9), reflux (Q2–3), indigestion (Q4–6 & 8), diarrhea (Q11–12 &14), constipation (Q10, 13 &15).

#### Safety assessment (blood samples)

16 ml fasting blood samples were collected before and after the supplementation period at visit 2 (baseline), visit 3 and visit 4 to determine the levels of sodium, potassium, chloride, urea & creatinine, bilirubin (total), bilirubin (direct), alanine aminotransferase (ALT), alkaline phosphatase (ALP), aspartate aminotransferase (AST), gamma-glutamyl transferase (GGT), total protein, albumin, globulin, calcium, magnesium & phosphate, uric acid, cholesterol, triglycerides, and glucose.

### Statistical analyses

All statistical analyses were carried out using SPSS Version 22 for Windows. The analyses variables were the changes in ^13^C-UBT values and the differences in GSRS score from baseline (day 1) to visit 3 (week 4) and from visit 3 to the end of the study (week 8).

Exploratory data analyses were conducted for the overall population and for each study period to determine statistically significant variance between the two different supplementations (placebo vs Pylopass™) for each endpoint assessed. Quantitative parameters and their changes were characterised by means and standard deviations on statistical relevance within the supplementation phases. Due to the deviation from normal distribution differences within a defined supplementation phase (e.g. pre- vs post- supplementation) were tested by the non-parametric Wilcoxon test.

Differences were considered significant when compared to a 0.05 level of significance. Cohen’s *d* classification was used to measure the strength of any observed difference (standardised mean difference) – i.e., the effect size of a result, where 0.2 ≤ *d* < 0.5 is a small effect size; 0.5 ≤ *d* < 0.8 is a moderate effect size; *d* ≥ 0.8 is a large effect size.

## Results

### Demographic and basic clinical characteristics

115 subjects were screened for entry into the study of which 24 were enrolled (Fig. 1). Reasons for ineligibility were failure to satisfy the inclusion criteria and/or satisfying conditions of the exclusion criteria. There were no dropouts and the 24 randomized subjects completed the study. Confirmation of tablet intake was recorded by tablet count and subjects were instructed to bring the vial to count the unused product. Compliance was measured through pill counts (quantifying unused capsules). Overall the compliance was good and more than 90.2% of the chewable placebo tablets (at visit 3) and 88.1% of the verum tablets (at visit 4) were consumed.

Of the 24 subjects participating in the study, 54% (13) were female and 46% (11) were male, with a mean age of 42.4 (SD 12.7 years). The majority of subjects were Caucasian. One of the subjects was African. Details of the study population are given in Table 1. There were no significant changes from baseline in any of the characteristics listed in Table 1 at the end of the study.

**Table 1:** Baseline characteristics of the study subjects

	Mean ± SD	Range
Age (years)	42.4 ± 12.7	25–73
Weight (kg)^a^	73.7 ± 12.3	53.7–96
Height (cm)^a^	171.7 ± 6.7	158–182.5
Pulse (bpm)	68.6 ± 9.1	54–82
Systolic blood pressure (mm Hg)^b^	122.9 ± 17.6	97–164
Diastolic blood pressure (mm Hg)^b^	82.9 ± 12.0	56–105
Urease breath test (δ13C)	13.4 ± 14.4	2.4–75.0

^a^Anthropometric measurements were assessed using a Tanita digital scale with a height assessment attachment. ^b^Blood Pressure was measured using an Omron (M10-IT model) blood pressure monitor

### Reduction of *H. pylori* load

**Fig. 2:**
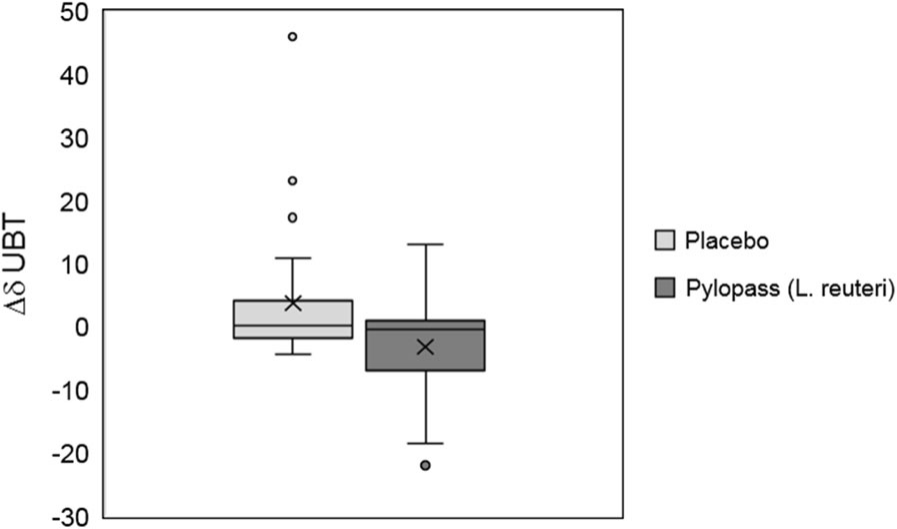
Change in ^13^C-Urea breath test (δUBT) calculated as absolute differences from baseline (day 1) to end of supplementation with placebo (day 28) and after application of Pylopass™ from day 29 to day 56 . The figure displays the results as medians (Day 28 – Day 1 and Day 56 – Day 28) with standard deviation. The respective means are marked with a cross. The FAS (full analysis set) study population consisted of *n* = 24 subjects

The primary endpoint of the study was the decrease in *H. pylori* colonization assessed via the urease breath test. Due to the large inter-individual variability of quantitative measures of colonization (^13^C UBT baseline), analysis of *H. pylori* load reduction by *L. reuteri* strain DSM 17648 (Pylopass™) was primarily based on intra-individual changes upon placebo and active supplementation (Δplacebo vs ΔPylopass™). Using this set of data, an increase (trend) in the ^13^C UBT value (average increase of 3.79 ± 11.2) from baseline (day 1) to day 28 (Fig. 2) is seen after placebo supplementation phase. In 13 (54.2%) of the subjects the placebo supplementation resulted in an increase of ^13^C UBT values by 37.3% (Table 2). In contrast, there was a trend for Pylopass™ supplementation to reduce the ^13^C UBT values (ΔPylopass™ -3.14 ± 8.2) from day 29 through day 56 (Fig. 2). In 15 (65.2%) of the subjects the *H. pylori* load decreased after Pylopass*™* supplementation by 22.5% (change in % of baseline). Responses showed some variability, from no reduction up to a delta of more than 20. A Wilcoxon test for differences showed that the difference of 3.14 in ^13^C UBT value between start and end of the Pylopass™ supplementation phase was statistically insignificant (*p* = 0.130, small effect size 0.38, power of 41.5%). However, by performing a power analysis of the current data it was found that increasing the sample size to 59 increased the probability of correctly finding a statistically significant decrease in *H. pylori* between the placebo and Pylopass™ groups, with an 80% power.

**Table 2:** Comparison of changes in *H. pylori* load between placebo and supplementation periods

Supplementation phase	Decrease in *H. pylori*	Increase in *H. pylori*
Baseline (day 1) – day 28	11 (45.8)	13 (54.2)
Day 29 – day 56	15 (62.5)	9 (37.5)

Number of subjects (%) who experienced a decrease/increase in *H. pylori* load as evaluated by the means of 13C UBT after placebo (day 1 -day 28) and after 4 weeks (day 29-56) of Pylopass*™* supplementation (*n* = 24)

### Reduction of gastrointestinal symptoms

This study, to our knowledge, is the first to evaluate the impact of *L. reuteri* DSM17648 containing Pylopass™ supplementation on mild gastric symptoms in *H. pylori* positive subjects.

For the secondary endpoint, the subjects were assessed at baseline, day 28 following the placebo phase and day 56 (following the Pylopass™ phase) of the supplementation period using the Gastrointestinal Symptom Rating Scale (GSRS) questionnaire, which comprises 15 questions in 5 categories (abdominal, reflux, indigestion, diarrhea, and constipation).

**Fig. 3:**
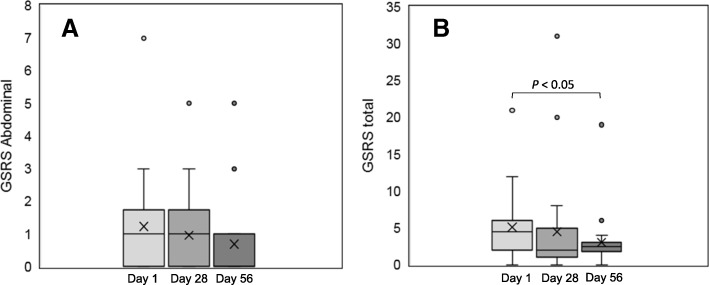
Gastrointestinal Symptom Rating Scale (GSRS) scores for abdominal symptoms **(a)** and total GSRS scores (**b**) at baseline (day 1), after 28 days placebo supplementation and after 28 days Pylopass™ supplementation. The figure displays the results as medians with standard deviation, the means are indicated with cross. The difference from baseline (day 1) to end off supplementation (day 56) was statistically significant (*p* = 0.05)

The overall gastrointestinal symptom rating scale (overall GSRS) and the results on the subcategories are summarized in Table 3. The overall GSRS significantly decrease during the study and highlighted an overall improvement. Total GSRS score was reduced from mean value of 5.16 to 4.5 after the placebo period from baseline (*p* = 0.085) and from 4.5 to 3.04 following verum supplementation (*p* = 0.200). The biggest decrease, although not statistically significant, was determined in the abdominal GSRS scores, where a decrease of 16.7% was measured. The values decreased from day 1 (baseline) through day 56 (endpoint) from 1.25 to 0.75 (Table 3). In the subcategories “indigestion” and “constipation” GSRS scores also decreased from baseline (day 1) following both placebo and Pylopass™ supplementation (e.g. day 1 through day 28 to day 56 reduced from 1.79 to 1.67 to 1.17 and from 0.79 to 0.71 and to 0.29, respectively; Table 3). While the reflux GSRS decreased from day 1 (baseline) to day 28 and remained constant from day 29 to day 56, the diarrhea GSRS remained constant throughout the study. In Fig. 3 the reduction of the median rates of abdominal GSRS (A) and total GSRS scores (B) are shown throughout the study. The differences in the GSRS values between the two supplementation phases are statistically insignificant for all categories.

**Table 3:** Gastrointestinal Symptom Rating Scale (GSRS) score at baseline (day 1), after 4 weeks of placebo supplementation (day 28) and after 4 weeks of *Pylopass™* supplementation (day 56)

GSRS (Score)	Day 1	Day 28	*p*-value	Day 56	*p*-value
GSRS total					
mean	5.16	4.50		3.04	
median	4.50	2.00	0.085	2.5	0.200
SD	4.61	6.98		3.79	
Abdominal					
mean	1.25	1.00		0.75	
median	1.00	1.00	0.182	0	0.217
SD	1.57	1.25		1.26	
Reflux					
mean	1.00	0.54		0.58	
median	1.00	0.00	0.062	0.00	0.904
SD	1.25	1.06		0.88	
Indigestion					
mean	1.79	1.67		1.17	
median	1.00	0.50	0.419	1.00	0.270
SD	1.77	2.81		2.08	
Diarrhoea					
mean	0.25	0.58	0.161	0.25	0.518
median	0.00	0.00		0.00	
SD	0.85	1.64		0.61	
Constipation					
mean	0.79	0.71		0.29	
Median	0.00	0.00	0.926	0.00	0.142
SD	1.32	1.43		0.46

Wilcoxon Test for differences showed that there was a statistically insignificant difference in overall gastrointestinal symptom rating scale (overall GSRS) of 3.24% between the placebo phase and the Pylopass™ supplementation phase (*p* = 0.200; small effect size 0.331). A power analysis of the current data demonstrated that increasing the sample size to 77 increased the probability of correctly finding a statistically significant decrease in *overall GSRS* between the placebo and Pylopass™ supplementation phases, with 80% power.

While there was not a statistically significant difference in overall GSRS score between the end of the Pylopass™ phase and the end of the placebo phase, there was a statistically significant decrease of 4.63% in overall GSRS scores between baseline and the end of the Pylopass™ phase, following the placebo phase (day 1 through 56; *p* = 0.005, moderate effect size = 0.733; Fig. 3b).

#### Safety assessment

There were no statistically significant alterations in a complete blood cell profile or in circulating metabolic enzymes with markers of liver and renal function measured as safety parameters before and after the supplementation period. During the course of study no changes in lifestyle, or general health were reported.

## Discussion

This placebo-controlled, single-blind study using a prolonged supplementation period of 28 (instead of 14) days, demonstrates a trend for *Lactobacillus reuteri* DSM17648 to both reduce *H. pylori* load and confer a beneficial effect on mild gastrointestinal symptoms in volunteers carrying *H. pylori*. After the 4-week supplementation phase with *L. reuteri* DSM17648 containing Pylopass™ there was a trend for *H. pylori* load reduction in 62.5% of the subjects, confirming the results of previous findings [15, 16]. The overall gastro-symptom rating scale (GSRS) scores have not been tested in previous studies using *L. reuteri* DSM17648. This study showed a trend for a decrease in 66.7% of subjects when they took *L. reuteri* DSM17648, with the biggest decrease occurring in the abdominal symptoms subgroup where GSRS scores decreased by 16.7%. This study design was exploratory in nature, and while a significant effect of Pylopass™ was not found on the reduction of *H. pylori* load or GSRS symptoms in the study population of 24 subjects, power calculations show that increasing the study size to *n* = 59 and *n* = 77, respectively, increased the chance of finding a statistically significant effect. Interestingly, there was a statistically significant decrease in GSRS scores on Day 56 compared to baseline (e.g. with the placebo phase preceding the Pylopass™ phase). Further human studies with adequate statistical power are necessary to confirm a significant effect of *L. reuteri* DSM17648 on *H. pylori* load and mild gastrointestinal symptoms in humans carrying *H. pylori*.

The projected significant decrease of the ^13^C-UBT δ (and thereby the intragastrical urease activity) is an important finding considering the substantial evidence reported in literature regarding the impact of *H. pylori* density on gastric inflammation, gastroduodenal endoscopic lesions and the development of severe complications. A number of studies have reported a significant correlation between the ^13^C-UBT and *H. pylori* bacterial load [17, 18] grade of gastritis activity [19], and gastric mucosal myeloperoxidase activity that is a quantitative marker of gastrointestinal inflammation [20].

Many studies have shown that high bacterial loads are associated with increased acute mucosal damage and long-term changes in the gastric mucosa, and further, the influence of *H. pylori* density reduction on improvement of gastric mucosal changes [21]. Substances like bismuth or the muco-protective drug teprenone used in various combinations or monotherapies, lead in most cases to a reduction of the bacterial load, which concomitantly resulted in a rapid and significant reduction of inflammation and gastritis activity [22, 23].

There are many factors influencing the severity of gastric inflammation such as *H. pylori* strain-specific virulence factors, host genetic factors, duration of infection, and subject age. Adverse clinical outcomes including peptic ulcer disease and gastric cancer are depending on a gentle balance between a harmless inflammation and a more severe inflammation. Eradication of *H. pylori* is the traditional and to date most effective way to avoid *H. pylori*-related complications; however, as the efficacy of eradication therapy is rapidly decreasing [24], the development of substances that reduce density and virulence of *H. pylori* such as the *L. reuteri* DSM17648 containing supplement Pylopass™ will be valuable strategies to prevent or reduce *H. pylori*-associated diseases. Application of a microbiological active like *L. reuteri* is promising when aiming at achieving reduction of bacterial levels, controlling inflammation, modulating the immune response, or inhibiting adherence of *H. pylori* to the gastric epithelium by reducing its motility. Such microbial solutions could be promising as an adjunct therapy for the current *H. pylori* treatment to achieve higher eradication rates [25, 26].

## Conclusion

The results demonstrated that *L. reuteri* DSM17648 has the potential to suppress *H. pylori* infection, and may lead to an improvement of *H. pylori*-associated gastro intestinal symptoms. Further studies with adequate power should be performed.

## Abbreviations

13C-UBT: 13 Carbon Urease Breath Test
*GSRS*: Gastrointestinal Symptom Rating Scale
*H. pylori*: *Helicobacter pylori*
*L. reuteri*: *Lactobacillus reuteri*
NSAID: Non-Steroidal Anti-Inflammatory Drug
SD: Standard Deviation
